# Elaiophylin reduces body weight and lowers glucose levels in obese mice by activating AMPK

**DOI:** 10.1038/s41419-021-04264-9

**Published:** 2021-10-20

**Authors:** Ruoxuan Bao, Yongmei Meng, Haibo Zhang, Chen Yang, Wei Li, Cheng Zhang, Jinye Zhang, Renqiang Sun, Zengxia Li, Wei Jiang, Chensong Zhang, Changsheng Zhang, Hai-Xin Yuan, Yongjun Dang

**Affiliations:** 1 The Molecular and Cell Biology Research Lab of the Institutes of Biomedical Sciences and the School of Basic Medical Sciences, Fudan University, Shanghai, 200032 China; 2 College of Traditional Mongolian Medicine, Inner Mongolia Medical University, Mongolia, 010110 China; 3 Key Laboratory of Tropical Marine Bioresources and Ecology, Guangdong Key Laboratory of Marine Materia Medica, Institution of South China Sea Ecology and Environmental Engineering, South China Sea Institute of Oceanology, Chinese Academy of Sciences, Guangzhou, 510301 China; 4 Key Laboratory of Metabolism and Molecular Medicine, the Ministry of Education, Department of Biochemistry and Molecular Biology, School of Basic Medical Sciences, Fudan University, Shanghai, 200032 China; 5 Department of Medicinal Chemistry, China Pharmaceutical University, Nanjing, Jiangsu 210009 China; 6 State Key Laboratory for Cellular Stress Biology, School of Life Sciences, Xiamen University, Xiamen, Fujian 361102 China; 7 Center for Novel Target and Therapeutic Intervention, Institute of Life Sciences, Chongqing Medical University, Chongqing, 400016 China

**Keywords:** Cell biology, Drug discovery

## Abstract

Obesity is an epidemic affecting 13% of the global population and increasing the risk of many chronic diseases. However, only several drugs are licensed for pharmacological intervention for the treatment of obesity. As a master regulator of metabolism, the therapeutic potential of AMPK is widely recognized and aggressively pursued for the treatment of metabolic diseases. We found that elaiophylin (Ela) rapidly activates AMPK in a panel of cancer-cell lines, as well as primary hepatocytes and adipocytes. Meanwhile, Ela inhibits the mTORC1 complex, turning on catabolism and turning off anabolism together with AMPK. In vitro and in vivo studies showed that Ela does not activate AMPK directly, instead, it increases cellular AMP/ATP and ADP/ATP ratios, leading to AMPK phosphorylation in a LKB1-dependent manner. AMPK activation induced by Ela caused changes in diverse metabolic genes, thereby promoting glucose consumption and fatty acid oxidation. Importantly, Ela activates AMPK in mouse liver and adipose tissue. As a consequence, it reduces body weight and blood glucose levels and improves glucose and insulin tolerance in both ob/ob and high-fat diet-induced obese mouse models. Our study has identified a novel AMPK activator as a candidate drug for the treatment of obesity and its associated chronic diseases.

## Introduction

An alarming increase in obesity has been observed worldwide, bringing about huge economic and social burdens [1–3]. Obesity is induced by an imbalance between caloric ingestion and energy expenditure, and it is associated with multiple metabolic disorders such as insulin resistance, hyperglycemia, and dyslipidemia. Furthermore, obesity is a high-risk factor for many chronic diseases, such as ﻿cardiovascular disorders, ﻿type-2 diabetes, and certain cancers [4–6]. Although increasing numbers of studies have focused on the development of anti-obesity drugs, safe and effective ﻿pharmacological options for obesity treatment remain elusive [7].

AMP-activated kinase (AMPK) is a highly conserved ﻿serine–threonine kinase that exists as a heterotrimeric complex consisting of catalytic α-subunits and regulatory β- and γ-subunits [8]. AMPK senses the energy status in cells by directly binding to AMP, ADP, or ATP via adenine nucleotide-binding sites on the γ-subunit. An increase of the AMP/ATP or ADP/ATP ratio, which is indicative of energy-stress conditions in cells, leads to conformational change and AMPK activation [9]. In addition, the phosphorylation of the conserved threonine in the activation loop of AMPK by upstream kinases [e.g., liver-kinase B1 ﻿(LKB1), calcium/calmodulin-dependent kinase kinase 2 (CAMKK2 or CAMKKβ)] is essential for its activation [10, 11]. Upon activation, AMPK inhibits ATP-consuming biosynthetic pathways, such as protein synthesis, and switches on catabolic pathways, including glucose uptake, glycolysis, and fatty acid oxidation [12]. The role of AMPK ﻿as an energy thermostat makes it an attractive target for obesity and its related complications [13, 14].

Natural products have long been used as the main source of drug discovery for various diseases [15, 16]. Elaiophylin (Ela), originally extracted from ﻿*Streptomyces melanosporus*, belongs to ﻿a special family of 16-membered macrodiolides with C2 symmetry [17]. It exhibits remarkable biological activities such as ﻿anticancer, antiangiogenic, and immunosuppressive properties [18]. Recent studies have shown that Ela, as an autophagy inhibitor, exhibited antitumor activity in ovarian cancer cells and ﻿multiple myeloma cells [19, 20]. As autophagy is a process controlled by metabolic regulators, including AMPK and mTOR, we sought to explore whether Ela targets metabolic processes. Our results demonstrate that Ela shows a significant effect on fat loss in obese mice with no obvious toxicity. We further illustrate that Ela activates AMPK in mouse tissues, as well as multiple cell lines, by rapidly inducing energy-stress conditions in cells. Our findings suggest that Ela is a novel and potent AMPK activator, which may be a therapeutic candidate for the treatment of obesity and its related complications.

## Results

### Ela treatment induces fat loss in obese mice

**Fig. 1:**
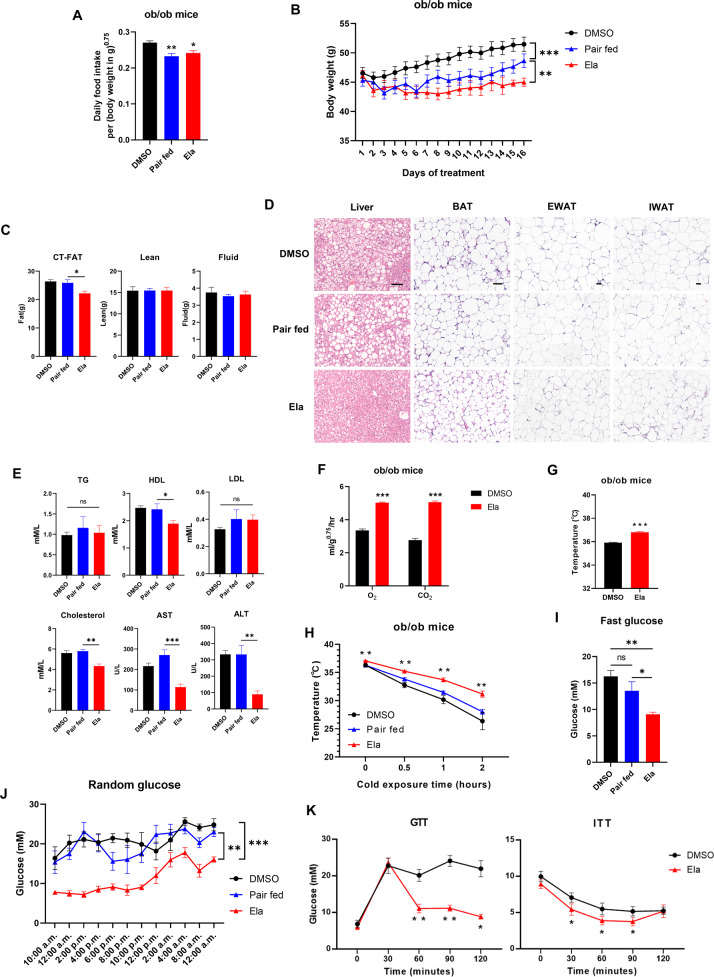
Ela treatment induces fat loss in ob/ob mice. **A** Food intake of ob/ob mice treated with DMSO or 5 mg/kg Ela. *n* = 6. **B** Body weight of ob/ob mice intraperitoneally injected with DMSO or Ela every three days, *n* = 6. **C** Body composition of ob/ob mice treated as in 1B for two weeks was measured by mice MRI system, *n* = 6. **D** Representative hematoxylin and eosin staining from liver, BAT, EWAT, and IWAT in ob/ob mice treated as in 1B for two weeks. BAT, brown-adipose tissue; IWAT, inguinal white-adipose tissue; EWAT, epididymal white adipose tissue. Scale bar, 50 μm. **E** Plasma triglyceride (TG), HDL, LDL, cholesterol, and AST and ALT were measured in ob/ob mice treated with DMSO or Ela for two weeks, *n* = 6. **F** Oxygen-consumption levels and CO_2_ production over a 24-h period in ob/ob mice treated with DMSO or Ela after two weeks, *n* = 6. **G, H** Rectal body temperature of ob/ob mice treated with DMSO or Ela for two weeks at room temperature or during cold exposure. Statistically significant differences in H were compared with pair-fed group, *n* = 6. **I, J** Fast and random glucose level of ob/ob mice treated as in 1B for two weeks. Fasting-glucose level recorded after a 6-h fast. Random glucose level was chased for 26 h *n* = 6. **K** Glucose tolerance test (GTT) and insulin-tolerance test (ITT) on ob/ob mice treated as in 1B after two weeks *n* = 6. Data are expressed as mean ± SEM. *P < 0.05, **P < 0.01, ***P < 0.001 for the indicated comparisons by one-way ANOVA with Tukey tests, except for **B**, **H**, **J**, **K**, two-way ANOVA and **F**, **G**, two-tailed unpaired Student’s *t*-test. ns = not significant.

To investigate whether Ela has any metabolic effect on obesity, we first used the obese ob/ob mouse model. Treatment of Ela showed a moderate reduction in food intake (Fig. 1a), so pair feeding was included in our study to eliminate the anorexigenic effect of Ela. Interestingly, although the pair-fed group showed body-weight reduction compared with the DMSO group, administration of Ela caused a larger extent of weight loss, and the effect was persistent during drug treatment (Fig. 1b). It is worth noting that body weight of pair-fed animals underwent fluctuation during the first one week, and then showed a steady rise during the rest experimental period, which indicated that these mice were adapted to mild fasting. Body-composition analysis revealed that the weight loss might have been due to inhibition of fat accumulation, but not lean or fluid (Fig. 1c). Consistently, Ela treatment significantly ameliorated steatosis in the obese mouse liver, as evidenced by the reduced accumulation of lipid droplets (Fig. 1d). In parallel, the size of adipocytes in brown adipose tissues (BAT), epididymal white-adipose tissues (EWAT), and inguinal white-adipose tissues (IWAT) was reduced by Ela (Fig. 1d). Nevertheless, Ela treatment lowered plasma levels of HDL and cholesterol, but not triglycerides or LDL (Fig. 1e). Moreover, blood ALT and AST levels were decreased by Ela treatment, indicating that fatty liver of obese mice was improved (Fig. 1e).

Next, we determined the actions of Ela on energy expenditure. During a 24-h light/dark cycle, mice with Ela treatment exhibited higher oxygen consumption and carbon dioxide production rates than the controls (Fig. 1f). Furthermore, a cold-tolerance test was performed to measure adaptive thermogenesis. Mice with Ela treatment had higher basal temperatures, indicating increased metabolism and thermogenesis (Fig. 1g). Consistently, when exposed to cold, body temperatures of ob/ob mice dropped quickly, whereas the Ela-treated group showed increased cold resistance (Fig. 1h), suggesting that Ela treatment improves adaption to cold exposure through thermogenesis.

Obesity is an important cause of insulin resistance and impaired glucose homeostasis [21]. Thus, we investigated whether Ela administration improves glucose ﻿parameters. Indeed, the levels of fasting glucose were decreased in mice treated with Ela (Fig. 1i). Moreover, the Ela-treated group showed decreased blood glucose level compared with the control and pair-fed groups during a 26-h tracing period (Fig. 1j). We also performed a glucose-tolerance test (GTT) and an insulin-tolerance test (ITT) in ob/ob mice, and observed that Ela treatment significantly lowered blood glucose levels in both tests (Fig. 1k). Our results demonstrate that Ela treatment reduces body and fat weight, promotes respiration, and ameliorates glucose tolerance in obese mice.

**Fig. 2:**
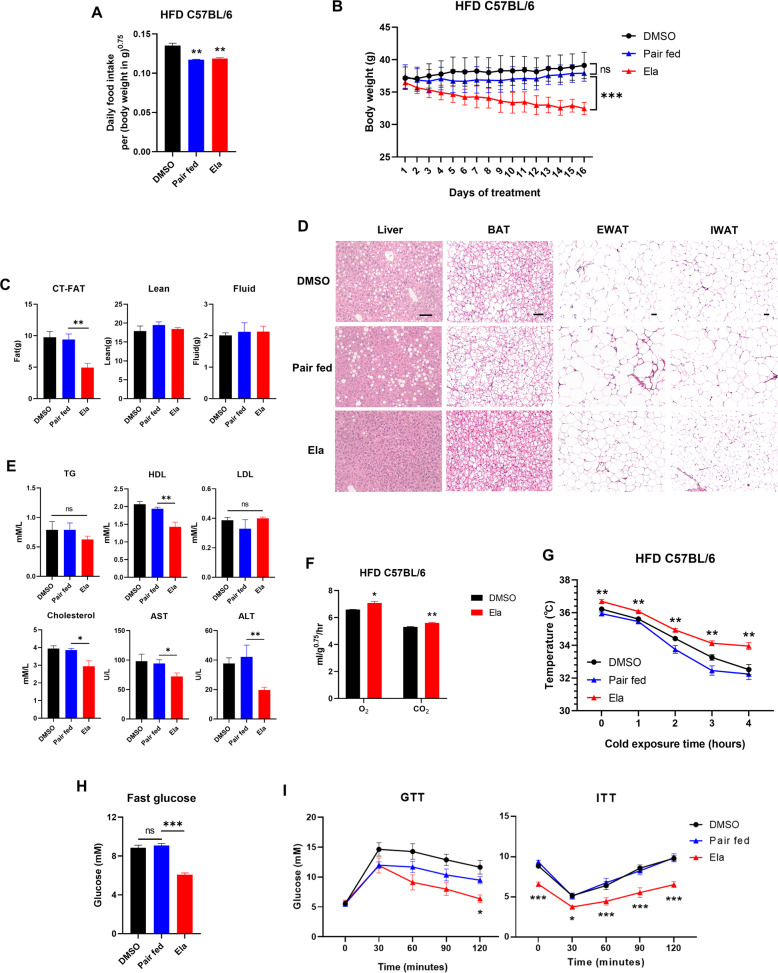
Ela treatment induces fat loss in diet-induced obese mice. **A** Food intake of HFD mice treated with DMSO or 5 mg/kg Ela, *n* = 6. **B** Body weight of HFD mice intraperitoneally injected with DMSO or Ela every three days, *n* = 6. **C** Body composition of HFD mice treated as in 2B for two weeks was measured by mice MRI system, *n* = 6. **D** Representative hematoxylin and eosin staining from liver, BAT, EWAT, and IWAT in HFD mice treated as in 2B for two weeks. BAT brown-adipose tissue, IWAT inguinal white-adipose tissue, EWAT epididymal white-adipose tissue. Scale bar, 50 μm. **E** Plasma triglyceride (TG), HDL, LDL, cholesterol, and AST and ALT were measured in HFD mice treated with DMSO or Ela for two weeks, *n* = 6. **F** Oxygen-consumption levels and CO_2_ production over a 24-h period in HFD mice treated with DMSO or Ela after two weeks, *n* = 6. **G** Rectal body temperature of HFD mice treated with DMSO or Ela for two weeks during cold exposure. Statistically significant differences were compared with pair-fed group, *n* = 6. **H** Fasting-glucose level recorded after a 6-h fast in HFD mice treated as in 2B for two weeks *n* = 6. **I** Glucose-tolerance test (GTT) and insulin-tolerance test (ITT) on HFD mice treated as in 2B after two weeks *n* = 6. Data are expressed as mean ± SEM. *P < 0.05, **P < 0.01, ***P < 0.001 for the indicated comparisons by one-way ANOVA with Tukey tests, except for **B**, **G**, **I**, two-way ANOVA and **F**, two-tailed unpaired Student’s *t*-test. ns = not significant.

To better mimic the pathological development of obesity, high-fat diet (HFD)-induced obese mouse model was used for Ela treatment. As observed in ob/ob mice, Ela treatment significantly decreased body weight with a slight reduction of food intake in HFD mice (Fig. 2a, b). Body composition, histological, and serum analysis further revealed inhibition in fat accumulation in Ela-treated HFD mice (Fig. 2c–e). As expected, mice with Ela treatment displayed enhanced energy expenditure and decreased blood glucose levels (Fig. 2f–i). Taken together, these results indicate that Ela has anti-obesity and glucose-lowering effects in the two obesity mouse models.

Drug treatment-related side effects are major challenges in medical translation. To evaluate the toxicity at the effective dose, wild-type C57BL/6 J mice were taken advantage as a nonobese model. Body weight and food intake were not affected by Ela treatment in nonobese mice (Supplementary Figs. 1a, b). Furthermore, histological morphology of the major organs and blood hemogram showed no statistical difference between Ela and DMSO groups (Supplementary Figs. 1c, d). Consistently, histological examination of vital organs and blood routine examination revealed no obvious toxicity of Ela treatment in both ob/ob and HFD mice (Supplementary Fig. 2, 3). Thus, our in vivo toxicity evaluation indicated that Ela is a potent and safe natural compound.

### Ela activates AMPK in the liver and adipose tissues

**Fig. 3:**
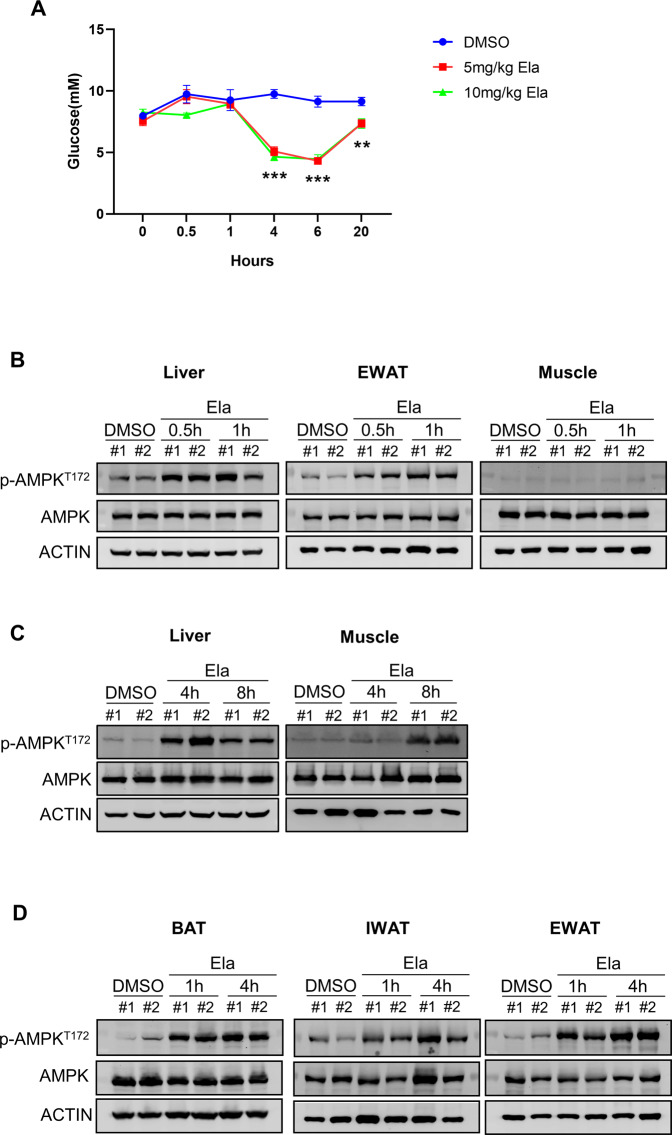
Ela activates AMPK in the liver and adipose tissues. **A** Wild-type C57BL/6 mice were intraperitoneally injected with DMSO, 5 mg/kg Ela or 10 mg/kg Ela. Blood glucose level was monitored at 0 h, 0.5 h, 1 h, 4 h, 6 h, and 20 h after injection *n* = 5. **B** Wild-type C57BL/6 mice were intraperitoneally injected with DMSO or Ela (80 mg/kg). Mice livers, EWAT, and muscles were harvested 0.5 h and 1 h after injection. WB analysis was performed with the indicated antibodies. **C** Wild-type C57BL/6 mice were intraperitoneally injected with DMSO or Ela (40 mg/kg). Mice livers and muscles were harvested 4 h and 8 h after injection. WB analysis was performed with the indicated antibodies. **D** Wild-type C57BL/6 mice were intraperitoneally injected with DMSO or Ela (80 mg/kg). Adipose tissues from different fat-pad depots were harvested 1 h and 4 h after injection. WB analysis was performed with the indicated antibodies. Data are expressed as mean ± SEM. **P < 0.01, ***P < 0.001 for the indicated comparisons by two-way ANOVA.

To explore the mechanism of Ela in lowering blood glucose levels, we performed a short-term Ela treatment study on wild-type C57BL/6 J mice. Similar to the observations in obese mice, intraperitoneal injection of Ela induced a rapid decrease in glucose levels at 1–6 h after Ela treatment, which gradually recovered to a normal level at 20 h after treatment (Fig. 3a). Mouse liver, EWAT, and muscle were harvested after Ela administration for subsequent analysis. Interestingly, AMPK, a key energy sensor and regulator, was rapidly activated by Ela within 0.5 h in the liver and EWAT, as evidenced by the increase in Thr172 phosphorylation on the AMPK-α subunit (Fig. 3b). However, AMPK activation was not observed within 1 h in the muscle. It might be caused by the inability of Ela to penetrate to the muscle within a short treatment period. Thus, we extended the Ela treatment time to 4 or 8 h and found that AMPK activation peaked at 4 h and declined at 8 h in the liver, while in the muscle, AMPK activation was only detected at 8 h after Ela treatment (Fig. 3c). Due to the effect of Ela on fat loss and the significant activation of AMPK in EWAT, we also tested other adipose tissues, including BAT and IWAT, for AMPK activation. Consistently, AMPK was rapidly activated within 1 h and remained activated until 4 h after Ela treatment (Fig. 3d).

### Ela activates AMPK in multiple cell lines

**Fig. 4:**
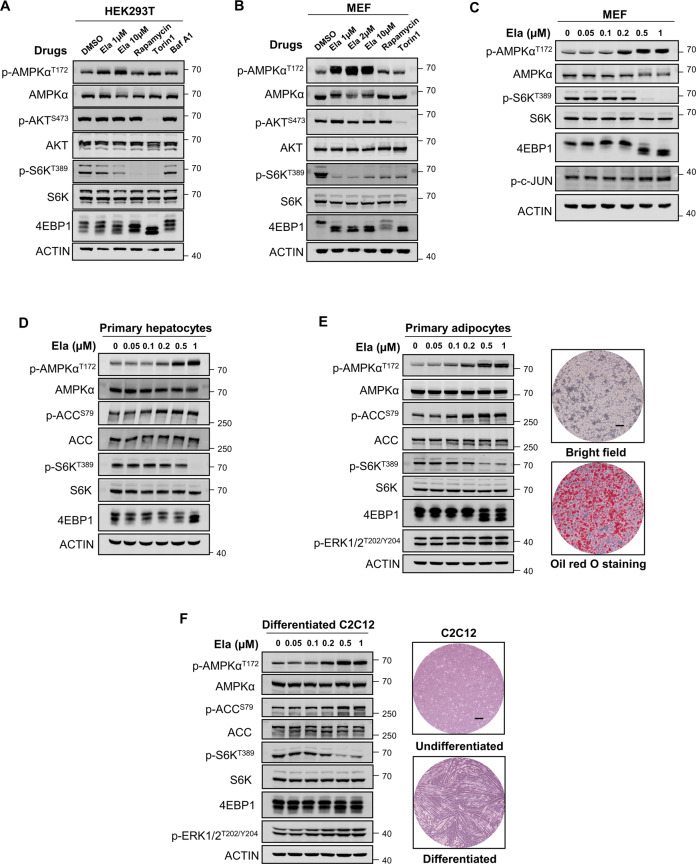
Ela activates AMPK in multiple cell lines. **A** HEK293T cells were treated with DMSO, Ela (1 μM), Ela (10 μM), mTORC1 inhibitor Rapamycin (0.1 μM), mTORC1/2 inhibitor Torin1 (0.1 μM), and autophagy inhibitor bafilomycin A1 (0.1 μM) for 3 h, then cells were harvested, and p-AMPKα^T172^, AMPKα, p-AKT^S473^, AKT, p-S6K^T389^, S6K, and 4EBP1 were detected by WB. **B** Mouse embryonic fibroblasts (MEFs) were treated with DMSO, Ela (1 μM), Ela (2 μM), Ela (10 μM), Rapamycin (0.1 μM), and Torin1 (0.1 μM) for 2 h, then cells were treated with Ela at the indicated concentration for 1 h. Later, cells were lysed and applied for WB. **C** Mouse embryonic fibroblasts (MEFs) were treated with Ela at the indicated concentration for 1 h, then cells were harvested and followed by WB. **D** Primary hepatocytes were isolated from wide-type C57BL/6 mice liver by two-step perfusion and then treated with Ela at the indicated concentration for 1 h. Cells were then lysed and followed by WB analysis. **E** Precursor cells were isolated from wide-type C57BL/6 mice inguinal white adipose tissue and induced to differentiation while the differentiation rate was shown by oil-red O staining. The mature primary adipocytes were treated with Ela at the indicated concentration for 1 h and followed by WB analysis. Scale bar, 100 μm. **F** Mouse myoblast C2C12 was induced to differentiation, and C2C12 myotubes were treated with Ela for the indicated concentration for 1 h. Cells were then harvested and applied for WB. Scale bar, 100 μm.

These results strongly suggest that Ela might induce metabolic changes by targeting AMPK. To verify this, we treated HEK293T and mouse embryonic fibroblasts (MEFs) with Ela, and then observed AMPK activation in a dose-dependent manner (Fig. 4a, b). AMPK and mTOR play antithetical roles in governing cellular metabolism, and AMPK can suppress mTORC1 activity by phosphorylating the TSC1/2 complex and Raptor subunit [22, 23]. Consistent with this, we observed suppression of mTORC1 activity in response to Ela treatment, as evidenced by the dephosphorylation of mTORC1 targets, S6K1 and 4EBP1 (Fig. 4a, b). By contrast, phosphorylation of Akt-Ser473, a target of the mTORC2 complex [24], was not affected by Ela treatment, which indicates that Ela only affects the AMPK–mTORC1 axis. It is notable that MEF cells are much more sensitive to Ela treatment. Therefore, we performed a downtitration experiment in MEF cells to identify the minimum effective dose of Ela. The results showed that Ela was able to activate AMPK at a concentration as low as 0.2 μM (Fig. 4c).

To further verify the effects of Ela on metabolic tissues, primary hepatocytes were subjected to Ela treatment. Similar to the observations in MEFs, Ela at concentrations greater than 0.2 μM promoted the phosphorylation of AMPK as well as its substrate acetyl coenzyme-A carboxylase (ACC) (Fig. 4d). In addition, we also isolated preadipocytes and induced adipogenic differentiation. Mature adipocytes were treated with Ela, which induced a similar AMPK activation as in hepatocytes (Fig. 4e). To validate Ela function in skeletal muscle, mouse C2C12 myoblasts were used as a model to mimic skeletal muscle [25]. Interestingly, Ela also significantly promoted the phosphorylation of AMPK and ACC at a dose comparable to that used in other cell studies (Fig. 4f). Collectively, the results suggest that Ela is a novel AMPK activator that can activate AMPK in multiple cell lines and primary cells.

### Ela activates AMPK through LKB1

**Fig. 5:**
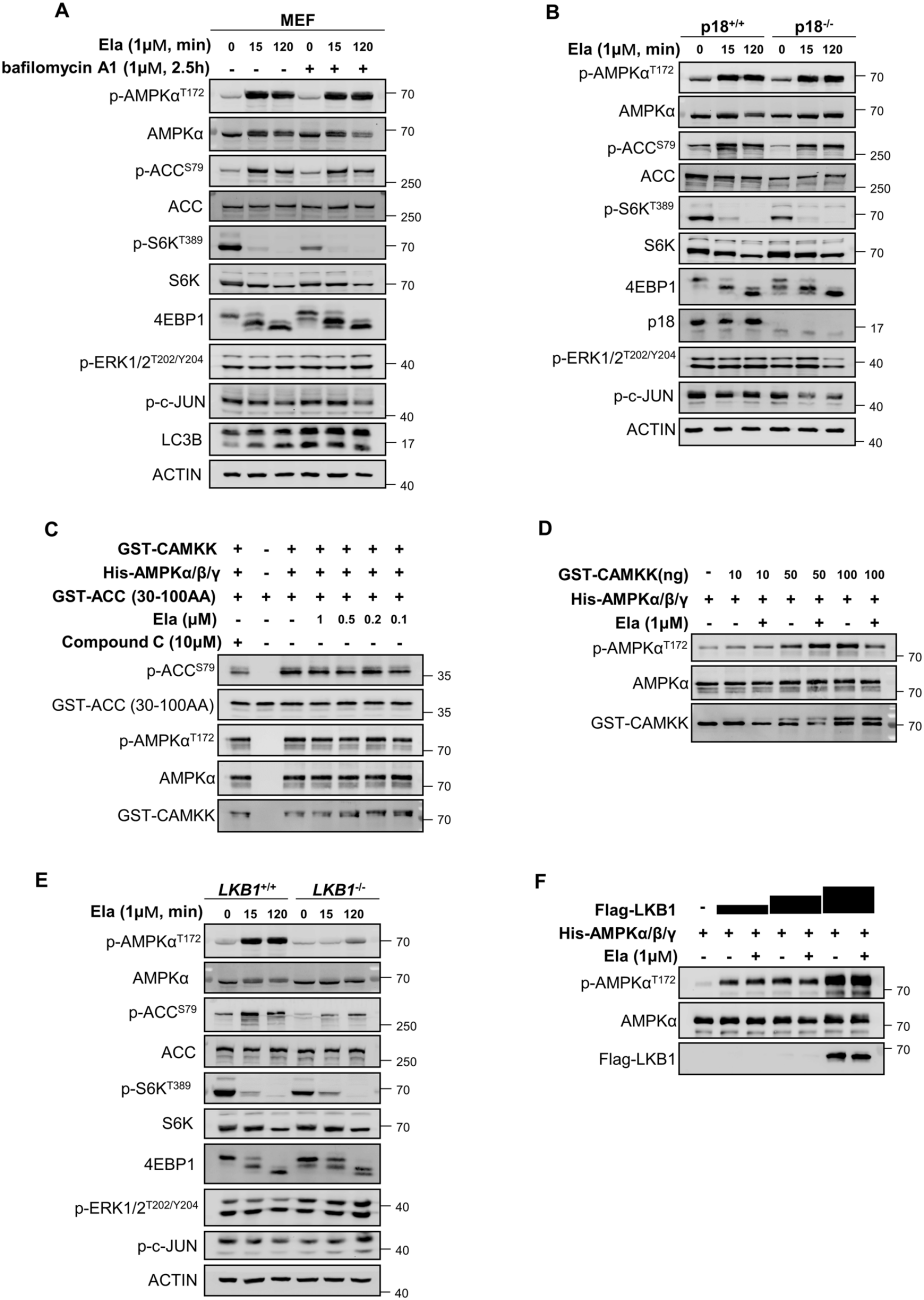
Ela activates AMPK through LKB1. **A** Mouse embryonic fibroblasts (MEFs) were pretreated with 1 μM bafilomycin A1 at first and then cells were treated with 1 μM Ela for the indicated time. Cells were then lysed and applied for WB. **B** p18^+/+^ and p18^−^^/^^−^ MEFs were treated with 1 μM Ela for the indicated time, then cells were harvested and followed by WB. **C** Purified GST-CAMKK, His-AMPK *α*/*β*/*γ*, and GST-ACC (30–100AA) were incubated with Ela at the indicated concentration in the kinase buffer for 30 min. The reaction was terminated by addition of SDS-loading buffer and was analyzed by WB. Compound C was used as a negative control. **D** Purified GST-CAMKK and His-AMPK *α*/*β*/*γ* were incubated with or without Ela in the kinase buffer for 30 min. The reaction was terminated by addition of SDS loading buffer and was analyzed by WB. **E** *LKB1*^+/+^ and *LKB1*^−/−^ MEFs were treated with 1 μM Ela for the indicated time, then cells were harvested and applied for WB. **F** Purified FLAG-LKB1 and His-AMPK α/β/γ were incubated with or without Ela in the kinase buffer for 30 min. The reaction was terminated by addition of SDS-loading buffer and was analyzed by WB.

The aforementioned observations prompted us to investigate the mechanism of AMPK activation by Ela. It was recently reported that the v-ATPase–Ragulator complex is essential for mTORC1 and AMPK docking and activation by the availability of glucose [26]. As Ela activates AMPK and inhibits mTORC1, we first explored whether it is required for this process. MEFs were pretreated with bafilomycin A1, an inhibitor of v-ATPase, followed by Ela administration. Bafilomycin A1 effectively promoted the conversion of LC3-I to LC3-II, a marker of autophagy [27], but had no influence on Ela-induced AMPK activation (Fig. 5a). Ela has been described as an autophagy inhibitor with long-term treatment [19], so we further explored the role of Ela on autophagy with different period of treatment. Interestingly, short-term Ela treatment led to increased conversion of LC3-II and p62 degradation (Supplementary Fig. 4a), indicating an increase of autophagy flux that resulted from AMPK activation. However, cells with long-term Ela treatment exhibited an increase of both p62 level and conversion of LC3-II (Supplementary Fig. 4b), which indicated autophagy inhibition and is consistent with previous studies [19, 20]. p18 (also known as LAMTOR1) is a membrane anchor of the v-ATPase–Ragulator complex on late endosomes/lysosomes [28]. We tested the effects of Ela in p18^+/+^ and p18^−/−^ MEFs, and observed that AMPK activation was identical in both cell lines (Fig. 5b). Taken together, these results suggest that Ela activates AMPK independent of lysosomal docking and signaling.

Then, we considered whether Ela could directly bind to and promote the kinase activity of AMPK. In vitro kinase assay was performed by using recombinant His-AMPK as the kinase and GST-ACC fragment as the substrate. In fact, no increase of ACC phosphorylation was observed with addition of Ela up to 1 μM (Fig. 5c). As a control, compound C, a known inhibitor of AMPK, compromised ACC phosphorylation by AMPK in vitro (Fig. 5c). Thus, Ela is unlikely to directly target the kinase activity of AMPK.

Activation of AMPK requires phosphorylation of the Thr172 site, which is mainly catalyzed by CAMKK2 or LKB1. We tested whether Ela can promote AMPK phosphorylation by upstream kinases. In vitro kinase assays showed that AMPK–T172 phosphorylation was elevated with increasing amounts of CAMKK2. However, Ela did not promote the activity of CAMKK2 toward AMPK (Fig. 5d). We also used the CAMKK inhibitor STO-609 or the Ca^2+^ chelator BAPTA-AM to pretreat cells, and observed that they did not affect Ela-induced AMPK activation (Supplementary Fig. 4c, d). These data indicate that CAMKK2 is not required for AMPK activation by Ela. Nonetheless, phosphorylation of AMPK and its target ACC was largely compromised in *LKB1*^−^^/−^ MEFs, compared with the normal activation in *LKB1*^+/+^ MEFs (Fig. 5e). Interestingly, Ela did not promote the activity of LKB1 toward AMPK–Thr172 as revealed by in vitro kinase assays (Fig. 5f). These results demonstrate that AMPK phosphorylation by LKB1 is essential for Ela-induced AMPK activation, but LKB1 is not a direct target of Ela.

### Ela induces energy stress by rapidly increasing AMP/ATP and ADP/ATP ratios

**Fig. 6:**
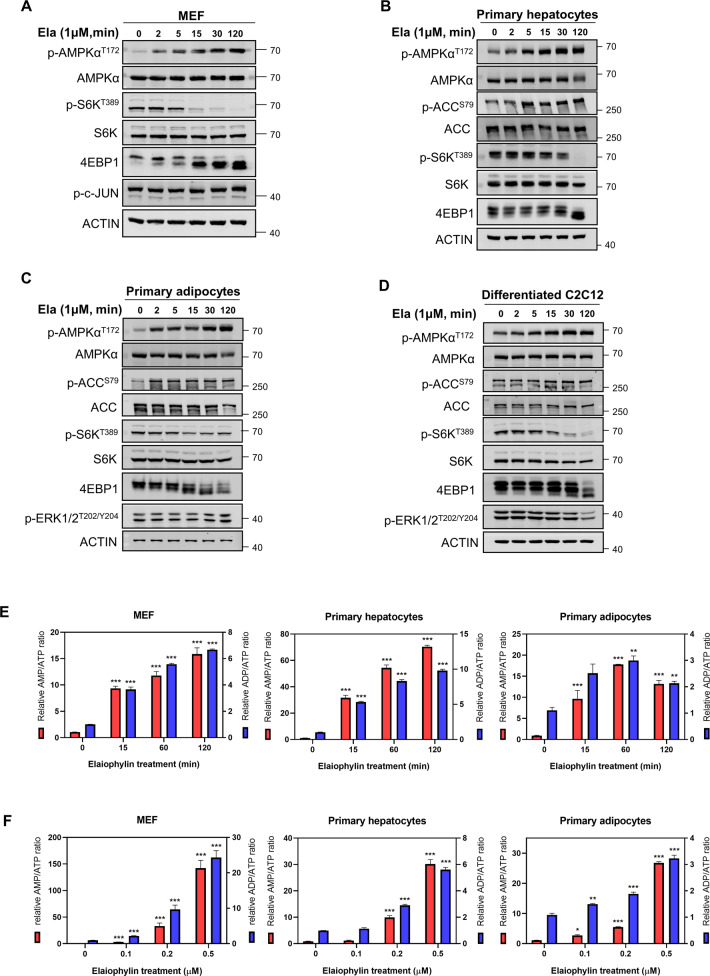
Ela induces energy stress by rapidly increasing AMP/ATP and ADP/ATP ratios. **A** Mouse embryonic fibroblasts (MEFs) were treated with 1 μM Ela for the indicated time, then cells were harvested and followed by WB. **B** Primary hepatocytes were treated with 1 μM Ela for the indicated time. Later, cells were harvested and applied for WB. **C** The mature primary adipocytes were treated with 1 μM Ela for the indicated time. Then cells were harvested and followed by WB. **D** C2C12 myotubes were treated with 1 μM Ela for the indicated time. Later, cells were harvested and applied for WB. **E** Mouse embryonic fibroblasts (MEFs), primary hepatocytes, and the mature primary adipocytes were treated with 0.5 μM Ela for indicated time. Intracellular AMP, ADP, and ATP levels were determined by LC–MS. Relative AMP/ADP and ADP/ATP ratio was shown. **F** Mouse embryonic fibroblasts (MEFs), primary hepatocytes, and the mature primary adipocytes were treated with Ela for 1 h at the indicated concentration. Intracellular AMP, ADP, and ATP levels were determined by LC–MS. Relative AMP/ADP and ADP/ATP ratio was shown. Data are expressed as mean ± SEM. *P < 0.05, **P < 0.01, ***P < 0.001 for the indicated comparisons by one-way ANOVA with Tukey tests.

To further explore how Ela activates AMPK, we performed an Ela time-course experiment. Interestingly, AMPK activation was observed as early as 2 min after Ela treatment in MEFs (Fig. 6a). Similar results were also observed in primary hepatocytes, adipocytes, and differentiated C2C12 cells, although the activation in C2C12 cells was a little slower than that in the other two cell types (Fig. 6b–d). Moreover, mTORC1 inhibition occurred significantly later than AMPK activation (Fig. 6a–d), further supporting the conclusion that mTORC1 inhibition is downstream of AMPK and Ela specifically targets the AMPK–mTORC1 axis. Cellular toxicity of Ela was examined by Western blot of apoptotic markers and PI/Annexin V staining and showed no obvious toxicity (Supplementary Fig. 5).

The rapid activation in AMPK by Ela treatment led us to hypothesize that Ela may induce rapid metabolic changes within cells. In addition to being phosphorylated by upstream kinases, cellular energy stress reflected by changes in the adenine-nucleotide level is also essential for AMPK activation [29]. For this reason, we measured AMP, ADP, and ATP levels in MEF, primary hepatocytes, and differentiated primary adipocytes. Interestingly, Ela treatment resulted in a rapid and robust increase in AMP/ATP and ADP/ATP ratios (Fig. 6e). This increase was sustained until 2 h, whereas a decrease was observed in adipocytes at 2 h. We also measured AMP/ATP and ADP/ATP ratios in response to different concentrations of Ela and found that both ratios were significantly elevated at a dose of 0.2 μM (Fig. 6f), which was identical to the effective dose of Ela on AMPK activation (Fig. 4). Collectively, it can be concluded that Ela is able to rapidly increase cellular AMP/ATP and ADP/ATP ratios, thereby leading to AMPK activation in a LKB1-dependent mechanism.

### Ela treatment accelerates glucose consumption and fatty acid oxidation

**Fig. 7:**
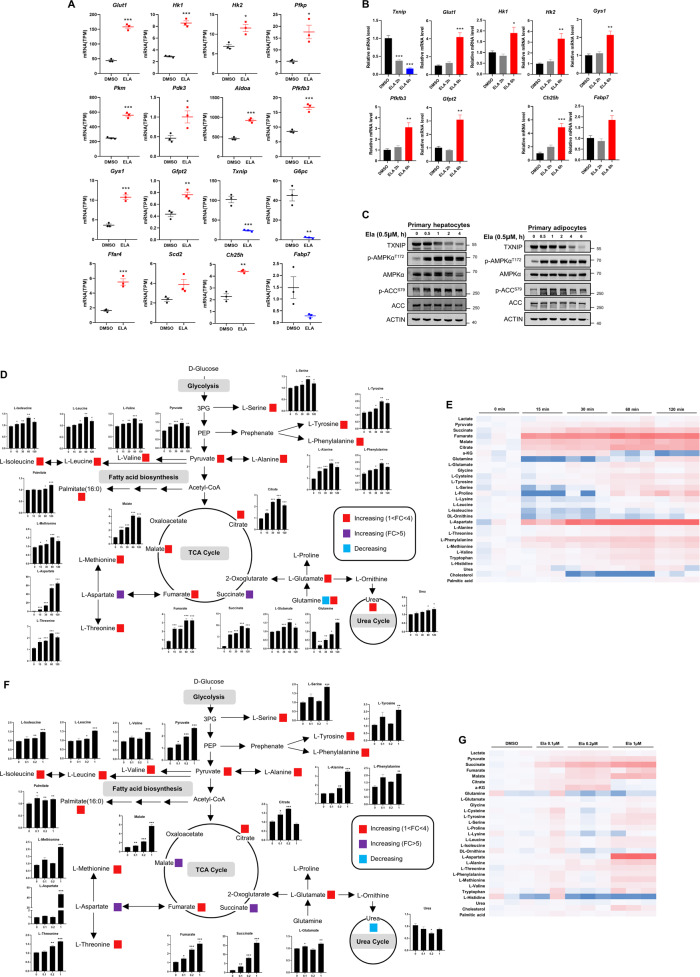
Ela treatment accelerates glucose consumption and fatty acid oxidation. **A** RNA-seq data of primary hepatocytes treated with DMSO or 0.5 μM Ela for 6 h showed that *Txnip* was downregulated, while *Glut1* and multiple metabolic enzymes involved in glucose consumption and fatty acid oxidation were upregulated by ELA treatment. **B** Primary hepatocytes were treated with DMSO or 0.5 μM Ela for the indicated time. mRNA levels of *Txnip*, *Glut1*, and several metabolic enzymes after treatment were quantified by qRT-PCR. **C** Primary hepatocytes and the mature adipocytes were treated with 0.5 μM Ela for the indicated time. Cells were then lysed and applied for WB. **D** Primary hepatocytes were treated with 0.5 μM Ela for the indicated time. Intracellular intermediates involved in glycolysis, TCA cycle, and amino acid metabolism were determined by GC–MS. The schematic diagram of metabolic flux that included those metabolites is shown. **E** Primary hepatocytes were treated with Ela at the indicated concentration for 1 h. Intracellular intermediates involved in glycolysis, TCA cycle and amino acid metabolism were determined by GC–MS. The schematic diagram of metabolic flux that included those metabolites is shown. **F, G** Heat map of metabolites detected in 3D and 3E. Data are expressed as mean ± SEM. *P < 0.05, **P < 0.01, ***P < 0.001 for the indicated comparisons by one-way ANOVA with Tukey tests, except for **A**, two-tailed unpaired Student’s *t*-test.

To further evaluate the impact of Ela-induced AMPK activation in the liver, we performed RNA-sequencing (RNA-seq) analysis on primary hepatocytes treated with or without Ela. Significant changes in a number of transcripts related to glucose consumption were observed by Ela treatment, including an upregulation of *Glut1* and a downregulation of *Txnip* (Fig. 7a), with the latter one reported to suppress glucose uptake [30]. Moreover, a number of glycolytic genes, such as *Hk1*, *Hk2*, and *Pfkp*, were upregulated (Fig. 7a). Ela treatment also resulted in increases in *Ffar4*, *Scd2*, and *Ch25h*, which was indicative of accelerated fatty acid oxidation (Fig. 7a). These expression changes were verified by quantitative RT-PCR (qRT-PCR) analysis (Fig. 7b). In addition, we also detected a decreased TXNIP protein level after Ela-induced AMPK activation in both primary hepatocytes and mature adipocytes (Fig. 7c).

To further investigate whether Ela promotes glucose consumption, we investigated the effects of Ela on metabolite changes of ﻿the tricarboxylic acid (TCA) cycle and amino acid metabolism in primary hepatocytes. Metabolic pathways and heat-map analyses are presented in Fig. 7d, e, respectively. Ela stimulated glucose into the TCA cycle, as the levels of TCA-cycle intermediates citrate, succinate, fumarate, and malate were increased. Among them, succinate showed the most significant fold change, which is approximately 10-fold. In addition, amino acid levels were promoted, as measured by the levels of serine, tyrosine, phenylalanine, valine, alanine, methionine, aspartate, and threonine. Aspartate showed the most dramatic increase with a fold change around 65. Moreover, the levels of the elevated metabolites gradually increased with Ela treatment within 1 h and decreased slightly after 2 h. We also observed that the metabolites involved in the TCA cycle and amino acid metabolism were elevated by Ela in a dose-dependent manner (Fig. 7f and g), which is consistent with AMPK activation by an Ela dose of 0.2 μM. Thus, these results demonstrate that Ela promotes glucose consumption and fatty acid oxidation.

## Discussion

Numerous studies employing genetic and obesity models have revealed a promising role for AMPK as a pharmacological target in the treatment of diverse metabolic disorders such as obesity, type-2 diabetes, and cardiovascular disease [31, 32]. Metabolic changes induced by AMPK through the regulation of fatty acid and glucose metabolism are beneficial in these conditions [33]. In this study, we uncovered Ela as a novel AMPK activator. In two obese mouse models, Ela functions as an effective compound in alleviating the clinical signs of obesity syndromes such as lowering glucose levels, inducing fat loss, and improving insulin sensitivity. Our results indicate that Ela can be a potential drug in the treatment of obesity.

The liver is vital for controlling circulating carbohydrate and fatty acid levels, and is critical in the development of obesity [34]. Multiple AMPK activators, such as A769662 [35] and the widely used diabetes medication metformin [36, 37], have been reported to inhibit hepatic lipogenesis, suppress glucose production, and improve insulin sensitivity. Here, we show that in addition to adipose tissues, Ela also exhibits remarkable functions in the liver by reducing blood glucose levels and improving glucose tolerance. Moreover, RNA-seq in primary hepatocytes indicated that Ela treatment accelerates glucose and lipid catabolism (Fig. 7). However, conditional knockout mice will be required to determine whether AMPK is the specific target of Ela, thereby leading to the beneficial effects in obese mice.

The mechanisms of AMPK activation have been extensively studied. In addition to the classical model that involves phosphorylation by upstream kinases and changes in adenine-nucleotide ratios, recent findings suggest that AMPK can directly sense glucose without energy change, a process that is mediated by fructose-1,6-bisphosphate (FBP) and aldolases [38]. This glucose-sensing mechanism occurs prior to the change in the energy status in cells, and requires the binding of AMPK to lysosomal membranes by p18, a component of the Ragulator complex [26]. However, our experiments in p18-knockout MEFs demonstrated that Ela induced rapid AMPK activation, which was independent of lysosomal translocation, although phosphorylation by upstream LKB1 was required (Fig. 5). Instead, Ela may rapidly activate AMPK by inducing a drastic increase in AMP/ATP and ADP/ATP ratios within 15 min, which was unlikely due to the inhibition of ATP synthesis in mitochondria. It can be speculated that Ela robustly promotes some energy-consuming processes or ATP hydrolysis, which is involved in molecular motors in cells [39]. Further work is expected to reveal the specific target(s) and mechanisms of Ela activity.

AMPK activation promotes catabolic processes to meet the energy requirements of cells. Consistently with Ela-induced AMPK activation, we observed an upregulation of genes involved in glycolysis and fatty acid oxidation, which was accompanied by an increase of metabolites involved in glucose metabolism and amino acid synthesis (Fig. 7). It is worth noting that the upregulation of most genes occurred much later (6 h after treatment) than AMPK activation induced by Ela, indicating that their expression may be affected by AMPK through indirect mechanisms. In fact, genes, such as GYS1 [40] and PFKFB3 [41, 42], are known to be regulated by AMPK at the protein level through direct phosphorylation. TXNIP is an α-arrestin family member that promotes GLUT1 internalization, thereby suppressing glucose uptake. AMPK is known to phosphorylate TXNIP and induce its rapid degradation [30]. Consistently, we observed a reduction in the TXNIP protein level after Ela-induced AMPK activation in primary hepatocytes and differentiated primary adipocytes. Interestingly, the mRNA level of TXNIP was also significantly reduced as early as 2 h after Ela treatment. Although the mechanism remains unclear, it is worthy to investigate whether the transcription of TXNIP is also regulated in an AMPK-dependent manner.

A recent study reported that long-term AMPK activation has adverse metabolic consequences, such as hyperphagia, obesity, and impaired insulin secretion, eliciting a caution for identifying AMPK activators [43]. Although AMPK activity remains high at 2 h after Ela treatment in most cells, except HCT116 cells in which p-AMPK decreased after 0.5 h (Fig. 6 and Supplementary Fig. 6), a reduction in AMP/ATP and ADP/ATP ratios was observed at 2 h in Ela-treated primary adipocytes (Fig. 6e). Moreover, many metabolites that were upregulated by short-term Ela treatment decreased at 2 h (Fig. 7d). This observation suggests that long-term Ela treatment may desensitize cells to AMPK activation, thereby compromising the potential adverse effects of Ela in cells. In support of this, in vivo and in vitro toxicity evaluation revealed no obvious toxicity under our experimental conditions (Supplementary Figs. 1–3, 5).

Collectively, we demonstrate that the novel AMPK activator Ela reduced body weight in obese mice by inducing fat loss and improving blood glucose balance. Nonetheless, much more work is needed for Ela to be developed into an anti-obesity drug. In addition, studies on Ela’s biological target, its analogs, and the medicinal modification of Ela may be of further interest.

## Materials and methods

### Chemicals and antibodies

Elaiophylin was prepared from the deep-sea-derived *Streptomyces* sp. SCSIO 1934 [44]. Rapamycin, torin1, bafilomycin A1, compound C, STO-609, and rosiglitazone were purchased from Selleck. Insulin, IBMX, and dexamethasone were purchased from Sigma-Aldrich. Antibodies recognizing phospho-AMPKα (#2535), AMPKα (# 2532), phospho-ACC (#3661), ACC (#3662), phospho-AKT (#4060), phospho-S6K (#9204), S6K (#9202), 4EBP1 (#9644), phospho-ERK (#4370), phospho-c-JUN (#3270), and LC3B (#3868) were procured from Cell Signaling. Other antibodies included anti-AKT (Proteintech, 60203-2-Ig), anti-GST (Proteintech, 10000-0-AP), anti-TXNIP (Abcam, ab188865), and anti-ACTIN (Genescript, A00702).

### Cell culture

HEK293T, HCT116, U2OS, HeLa, and C2C12 cells were obtained from the American Type Culture Collection (ATCC). WT, *p18*^−/−^, and *LKB1*^−/−^ MEFs were kindly provided by Dr. Lin Shengcai (Xiamen University). HEK293T, HeLa, C2C12, and MEF cells were cultured in Dulbecco’s modified Eagle’s medium (DMEM). HCT116 cells were cultured in McCoy’s 5a medium. U2OS cells were cultured in RPMI-1640 medium. All media were supplemented with 10% fetal bovine serum (Gibco) and 50 μg/ml penicillin/streptomycin. Cells were cultured at 37 °C in a 5% CO_2_ environment. HEK293T, U2OS, C2C12, HCT116, HeLa, and MEFs were tested for mycoplasma contamination and were negative.

### Immunoblotting

Homogenized cells and tissues were lysed in RIPA buffer and denatured in SDS-loading buffer containing dithiothreitol. Samples were loaded onto SDS-PAGE gels for electrophoresis. Proteins were transferred onto nitrocellulose membranes and immunoblotted with specific antibodies.

### qRT-PCR

Total RNA was prepared with the Trizol method and reversely transcribed with oligo-dT primers following the manufacturer’s instructions. Real-time PCR was performed with gene-specific primers in the presence of SYBR Premix Ex *Taq* (Takara) using the QuantStudio 6 Flex Real-Time PCR system (Applied Biosystems). Primer sequences are listed in Supporting Information Table S1.

### In vitro kinase assay

GST-ACC was expressed in the *E. coli* strain BL21 (DE3), induced with IPTG, and purified. For active LKB1 kinase, FLAG-tagged LKB1 was cotransfected with equimolar amounts of MO25 and STRAD-expression plasmids into HEK293T cells and immunoprecipitated by M2-agarose (Sigma) in NP-40 lysis buffer. Prior to kinase assays, immunoprecipitated LKB1 was washed three times with kinase buffer. Purified GST-CAMKK and His-AMPK α/β/γ were gifts from Dr. Lin Shengcai (Xiamen University). Kinase assays were carried out in kinase buffer (50 mM Tris-HCl [pH 7.4], 50 mM NaCl, 2 mM MgCl_2_, and 200 mM ATP) containing 200 mM AMP for 30 min at 30 °C. Reactions were terminated by the addition of SDS-loading buffer and analyzed by immunoblotting.

### Intracellular metabolite measurement

The relative intracellular levels of AMP, ADP, and ATP were measured by LC–MS. The abundance of other metabolites was detected by gas chromatography–mass spectrometry (GC–MS). Cells were washed with PBS and fixed by the immediate addition of 1 mL of prechilled (−80 °C) 80% (v/v) methanol. Metabolites were extracted by rotating at 4 °C for 1 h, followed by centrifugation at 13,000 g for 15 min at 4 °C. Supernatants were analyzed by ultra-high-performance liquid chromatography (Acquity, Waters) coupled to a Q Exactive hybrid quadrupole–orbitrap mass spectrometer (Thermo Fisher). For GC–MS, supernatants were dried and oximated with 30 μl of pyridine containing 20 mg/ml methoxyamine hydrochloride at 37 °C overnight and further derivatized with 20 μl of N-tert-butyldimethylsilyl-N-methyltrifluoroacetamide at 70 °C for 30 min. Samples were analyzed by Agilent 7890 A gas chromatography coupled with Agilent 5975 C mass spectrometer and analyzed by Analyst software (version 1.6).

### Hepatocyte isolation and culture

Hepatocytes were isolated from wild-type C57BL/6 mice using the two-step collagenase-perfusion method. Primary liver-cell suspensions were centrifuged at 50 g for 5 min. Cell pellets were obtained and suspended in DMEM containing 10% FBS. Media were changed to DMEM containing 2% FBS after 4–6 h to remove cell debris and cultured overnight.

### Induction of myogenic differentiation

C2C12 myoblasts at 80–90% confluence were induced to differentiate into myotubes by changing media to DMEM supplemented with 2% horse serum. Differentiation media were changed every two days for 6–8 days.

### Isolation of SVF cells and induction of adipogenesis

IWAT was harvested, and SVF cells were isolated by enzymatic digestion (collagenase VIII, Sigma). Digested tissues were filtered through a 100-μm mesh filter and centrifuged. Cell pellets were resuspended in ammonium chloride lysis buffer to remove red-blood cells. Preadipocytes were plated at low density and cultured in DMEM/F12 containing 10% FBS. Two days post confluency (designated day 0), cells were induced to differentiate with 10 μg/ml insulin (I), 1 μM dexamethasone (D), 0.5 mM 3-isobutyl-1-methylxanthine (M), and 1 μM rosiglitazone for two days. Cells were fed with DMEM/F12 supplemented with 10% FBS containing 10 μg/ml insulin and 1 μM rosiglitazone for two days, after which they were fed with DMEM/F12 containing 10% FBS for 2–4 days.

### Oil-red O staining

Cells were washed with PBS and fixed with 10% formalin for 30 min. Cells were washed twice with water, after which 60% isopropanol was added to the cells for 5 min. Thereafter, cells were stained with Oil Red O working solution for 20 min. Nuclei were stained with hematoxylin for 1 min. Cells were covered with water and viewed under a light microscope.

### Animal experiments

Mice were housed in pathogen-free facilities at Fudan University. All animal-related experimental procedures were performed in accordance with the National Institutes of Health guidelines and approved by the Laboratory Animal Ethical Committee of Fudan University. We complied with all relevant ethical regulations while conducting animal experiments and determined the number of mice required as the minimal number of mice necessary for reliable results. Adult male ob/ob mice and C57BL6/J mice at six weeks of age were purchased from SLAC (Shanghai).

For chronic anti-obesity studies, mice were fed a HFD for 10 weeks (60% calories from fat, 20% calories from protein, and 20% calories from carbohydrate; Research Diets). Ob/ob and HFD mice were matched by body weight, and randomly divided into three groups (6 mice/group) and injected intraperitoneally with Ela (5 mg/kg) every three days. Body weights and food intake were recorded every day throughout the experiment. In the pair-fed group, animals were fed with the same amount of food that was consumed by Ela group over the preceding 24 h. Body compositions were assessed using an NMR analyzer (Bruker). At the end of the experiment, BAT, IWAT, EWAT, and liver tissues were collected and fixed in 4% paraformaldehyde for hematoxylin and eosin staining. Blood samples were centrifuged within 30 min of collection to obtain plasma. Triglycerides, HDL, LDL, cholesterol, AST, and ALT levels were assayed using an automatic biochemical analyzer (Cobas c 311, Roche). No animals were excluded from the analysis. No blinding was performed in experimental mouse interventions as knowledge of the treatment groups was required.

### Cold-tolerance test

Mice were exposed to 4 °C with free access to water, but not food. Rectal temperatures were monitored using a rectal thermometer (PhysiTemp Instruments).

### GTT and ITT

Mice were fasted prior to tolerance tests. Glucose (2 mg/g body weight, for GTTs) or insulin (1 U/kg body weight, for ITTs) was injected intraperitoneally. Tail-blood glucose levels were measured using the Aviva ACCU-CHEK glucose meter post injection at the indicated times.

### Metabolic studies

Mice were maintained individually in a metabolism chamber (Comprehensive Lab Animal Monitoring System, CLAMS) with free access to water and food for 72 h. Thereafter, mice were housed for 24 h for adaption. Metabolic parameters, including O_2_ consumption and CO_2_ production, were recorded at 10-min intervals using a standard light–dark cycle at 25 °C.

### Statistical analysis

No statistical methods were used to predetermine sample size. Instead, they were chosen based on the literature and standard protocols in the field, as well as to ensure adequate power for the later statistical analyses. No data were excluded from the analyses.

The results were analyzed and graphed using Prism 8.0.1 software (Graphpad Software). All data shown represent the results obtained from at least three biological or technical replicates and are presented as mean ± SEM. All data meet the assumptions of the tests and the variance was similar between the compared groups. Comparisons between two groups were assessed by two-tailed unpaired *t*-test. Multiple comparisons versus the corresponding control groups were analyzed by one-way analysis of variance (ANOVA). Two-way ANOVA was used to examine interactions between variables. A *P*-value <0.05 was considered statistically significant.

## Supplementary information

Table S1

supplementary information

## Data Availability

RNA-seq files can be found under GEO accession number GSE182566.
