# Supplementary material for: Elaiophylin reduces body weight and lowers glucose levels in obese mice by activating AMPK
Source: Cell Death Dis. 2021 Oct 20;12(11):972. doi: 10.1038/s41419-021-04264-9 (PMC8528873; doi:10.1038/s41419-021-04264-9)
Supplement: Supplementary file 1 — Table S1 [file 41419_2021_4264_MOESM1_ESM.docx]

**Supplementary Table 1**. Primers for qRT-PCR

| Name | Primer |
| --- | --- |
| *Txnip*-F | TCTTTTGAGGTGGTCTTCAACG |
| *Txnip*-R | GCTTTGACTCGGGTAACTTCACA |
| *Glut1-*F | CAGTTCGGCTATAACACTGGTG |
| *Glut1*-R | GCCCCCGACAGAGAAGATG |
| *Hk1*-F | CGGAATGGGGAGCCTTTGG |
| *Hk1*-R | GCCTTCCTTATCCGTTTCAATGG |
| *Hk2*-F | TGATCGCCTGCTTATTCACGG |
| *Hk2*-R | AACCGCCTAGAAATCTCCAGA |
| *Gys1*-F | GAACGCAGTGCTTTTCGAGG |
| *Gys1*-R | CCAGATAGTAGTTGTCACCCCAT |
| *Pfkfb3*-F | CCCAGAGCCGGGTACAGAA |
| *Pfkfb3*-R | GGGGAGTTGGTCAGCTTCG |
| *Gfpt2*-F | ATGTGCGGAATCTTTGCCTAC |
| *Gfpt2*-R | GTCATAGCCCCTGTACTCCAG |
| *Ch25h*-F | TGCTACAACGGTTCGGAGC |
| *Ch25h*-R | AGAAGCCCACGTAAGTGATGAT |
| *Fabp7*-F | GGACACAATGCACATTCAAGAAC |
| *Fabp7*-R | CCGAACCACAGACTTACAGTTT |
| *Actin*-F | GGCTGTATTCCCCTCCATCG |
| *Actin*-R | CCAGTTGGTAACAATGCCATGT |
