# Supplementary material for: Elaiophylin reduces body weight and lowers glucose levels in obese mice by activating AMPK
Source: Cell Death Dis. 2021 Oct 20;12(11):972. doi: 10.1038/s41419-021-04264-9 (PMC8528873; doi:10.1038/s41419-021-04264-9)
Supplement: Supplementary file 2 — supplementary information [file 41419_2021_4264_MOESM2_ESM.pptx]

## Slide 1
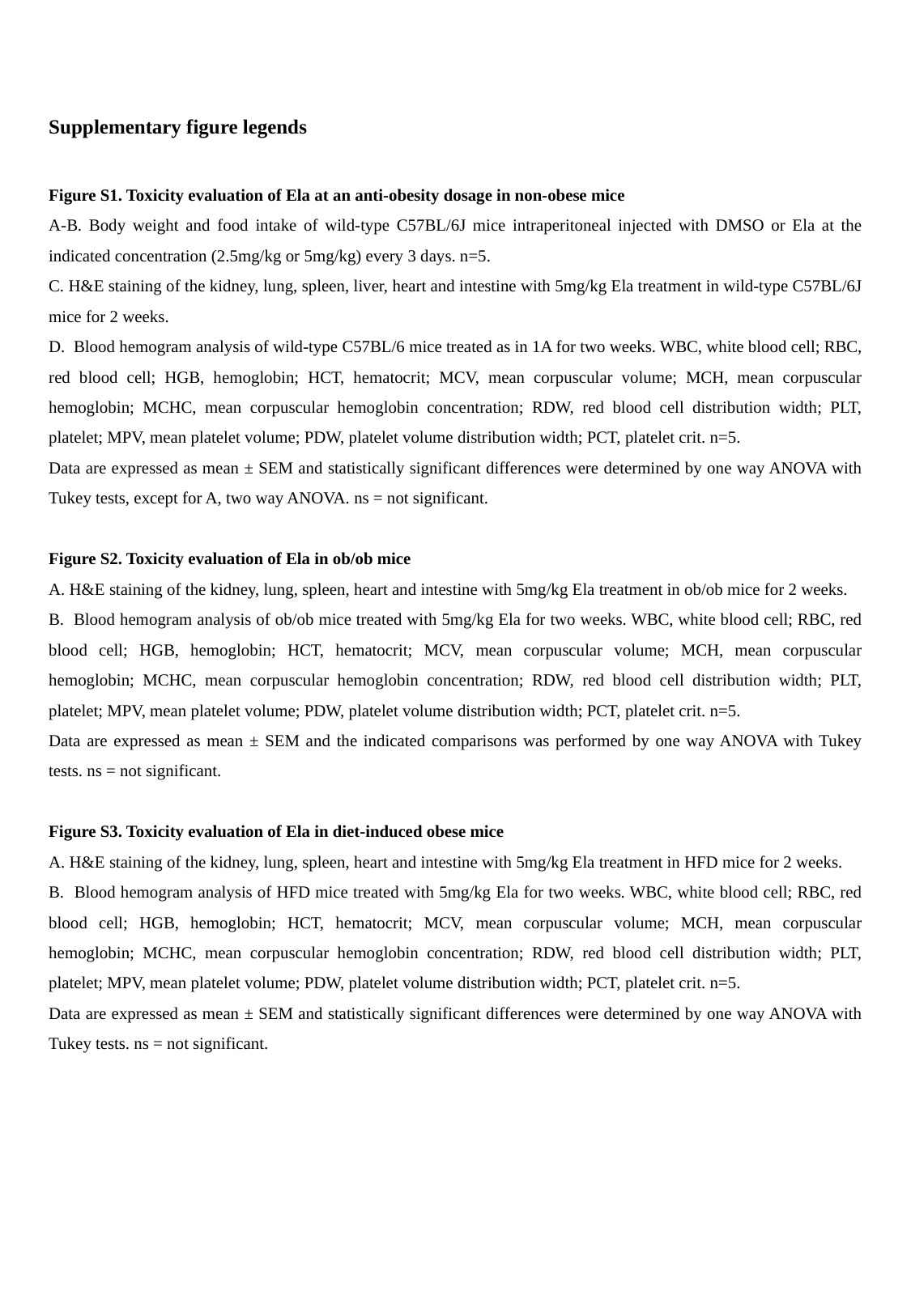

Supplementary figure legends
Figure S1. Toxicity evaluation of Ela at an anti-obesity dosage in non-obese mice
A-B. Body weight and food intake of wild-type C57BL/6J mice intraperitoneal injected with DMSO or Ela at the indicated concentration (2.5mg/kg or 5mg/kg) every 3 days. n=5.
C. H&E staining of the kidney, lung, spleen, liver, heart and intestine with 5mg/kg Ela treatment in wild-type C57BL/6J mice for 2 weeks.
D. Blood hemogram analysis of wild-type C57BL/6 mice treated as in 1A for two weeks. WBC, white blood cell; RBC, red blood cell; HGB, hemoglobin; HCT, hematocrit; MCV, mean corpuscular volume; MCH, mean corpuscular hemoglobin; MCHC, mean corpuscular hemoglobin concentration; RDW, red blood cell distribution width; PLT, platelet; MPV, mean platelet volume; PDW, platelet volume distribution width; PCT, platelet crit. n=5.
Data are expressed as mean ± SEM and statistically significant differences were determined by one way ANOVA with Tukey tests, except for A, two way ANOVA. ns = not significant.
Figure S2. Toxicity evaluation of Ela in ob/ob mice
A. H&E staining of the kidney, lung, spleen, heart and intestine with 5mg/kg Ela treatment in ob/ob mice for 2 weeks.
B. Blood hemogram analysis of ob/ob mice treated with 5mg/kg Ela for two weeks. WBC, white blood cell; RBC, red blood cell; HGB, hemoglobin; HCT, hematocrit; MCV, mean corpuscular volume; MCH, mean corpuscular hemoglobin; MCHC, mean corpuscular hemoglobin concentration; RDW, red blood cell distribution width; PLT, platelet; MPV, mean platelet volume; PDW, platelet volume distribution width; PCT, platelet crit. n=5.
Data are expressed as mean ± SEM and the indicated comparisons was performed by one way ANOVA with Tukey tests. ns = not significant.
Figure S3. Toxicity evaluation of Ela in diet-induced obese mice
A. H&E staining of the kidney, lung, spleen, heart and intestine with 5mg/kg Ela treatment in HFD mice for 2 weeks.
B. Blood hemogram analysis of HFD mice treated with 5mg/kg Ela for two weeks. WBC, white blood cell; RBC, red blood cell; HGB, hemoglobin; HCT, hematocrit; MCV, mean corpuscular volume; MCH, mean corpuscular hemoglobin; MCHC, mean corpuscular hemoglobin concentration; RDW, red blood cell distribution width; PLT, platelet; MPV, mean platelet volume; PDW, platelet volume distribution width; PCT, platelet crit. n=5.
Data are expressed as mean ± SEM and statistically significant differences were determined by one way ANOVA with Tukey tests. ns = not significant.

## Slide 2
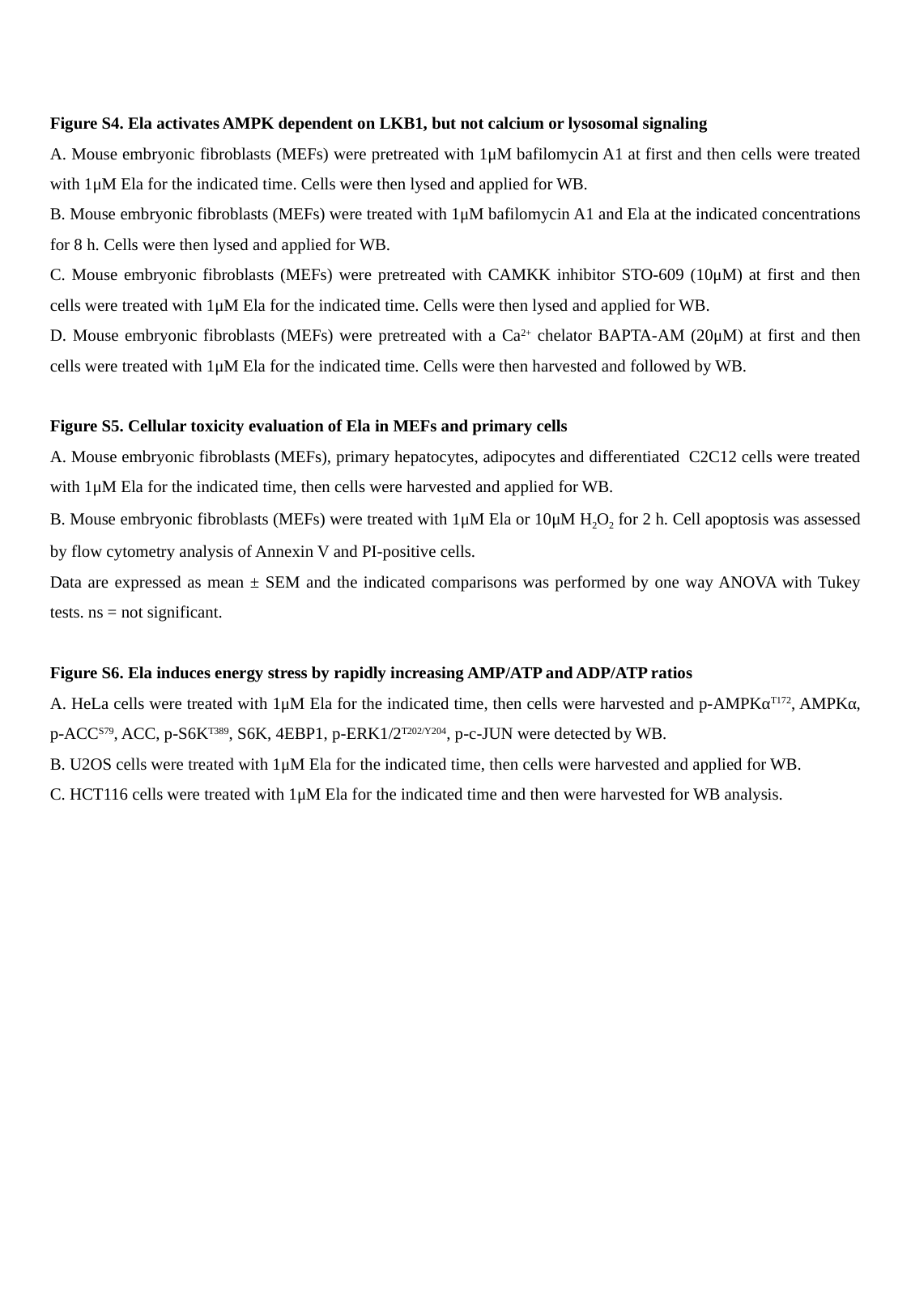

Figure S4. Ela activates AMPK dependent on LKB1, but not calcium or lysosomal signaling
A. Mouse embryonic fibroblasts (MEFs) were pretreated with 1μM bafilomycin A1 at first and then cells were treated with 1μM Ela for the indicated time. Cells were then lysed and applied for WB.
B. Mouse embryonic fibroblasts (MEFs) were treated with 1μM bafilomycin A1 and Ela at the indicated concentrations for 8 h. Cells were then lysed and applied for WB.
C. Mouse embryonic fibroblasts (MEFs) were pretreated with CAMKK inhibitor STO-609 (10μM) at first and then cells were treated with 1μM Ela for the indicated time. Cells were then lysed and applied for WB.
D. Mouse embryonic fibroblasts (MEFs) were pretreated with a Ca2+ chelator BAPTA-AM (20μM) at first and then cells were treated with 1μM Ela for the indicated time. Cells were then harvested and followed by WB.
Figure S5. Cellular toxicity evaluation of Ela in MEFs and primary cells
A. Mouse embryonic fibroblasts (MEFs), primary hepatocytes, adipocytes and differentiated C2C12 cells were treated with 1μM Ela for the indicated time, then cells were harvested and applied for WB.
B. Mouse embryonic fibroblasts (MEFs) were treated with 1μM Ela or 10μM H2O2 for 2 h. Cell apoptosis was assessed by flow cytometry analysis of Annexin V and PI-positive cells.
Data are expressed as mean ± SEM and the indicated comparisons was performed by one way ANOVA with Tukey tests. ns = not significant.
Figure S6. Ela induces energy stress by rapidly increasing AMP/ATP and ADP/ATP ratios
A. HeLa cells were treated with 1μM Ela for the indicated time, then cells were harvested and p-AMPKαT172, AMPKα, p-ACCS79, ACC, p-S6KT389, S6K, 4EBP1, p-ERK1/2T202/Y204, p-c-JUN were detected by WB.
B. U2OS cells were treated with 1μM Ela for the indicated time, then cells were harvested and applied for WB.
C. HCT116 cells were treated with 1μM Ela for the indicated time and then were harvested for WB analysis.

## Slide 3
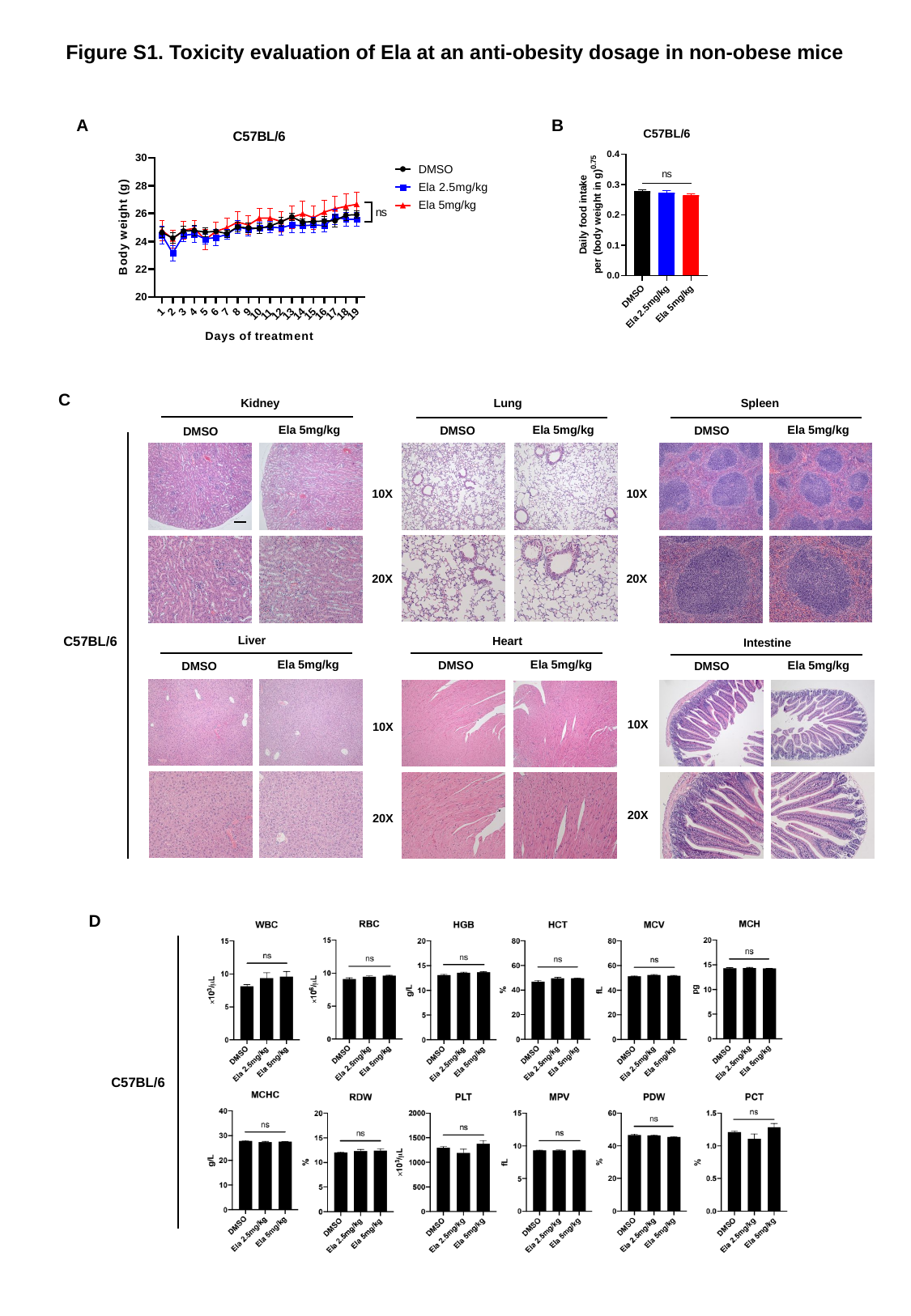

Figure S1. Toxicity evaluation of Ela at an anti-obesity dosage in non-obese mice
A
B
C
Spleen
Kidney
Lung
Ela 5mg/kg
Ela 5mg/kg
Ela 5mg/kg
DMSO
DMSO
DMSO
10X
10X
20X
20X
C57BL/6
Liver
Heart
Intestine
Ela 5mg/kg
Ela 5mg/kg
DMSO
Ela 5mg/kg
DMSO
DMSO
10X
10X
20X
20X
D
C57BL/6

## Slide 4
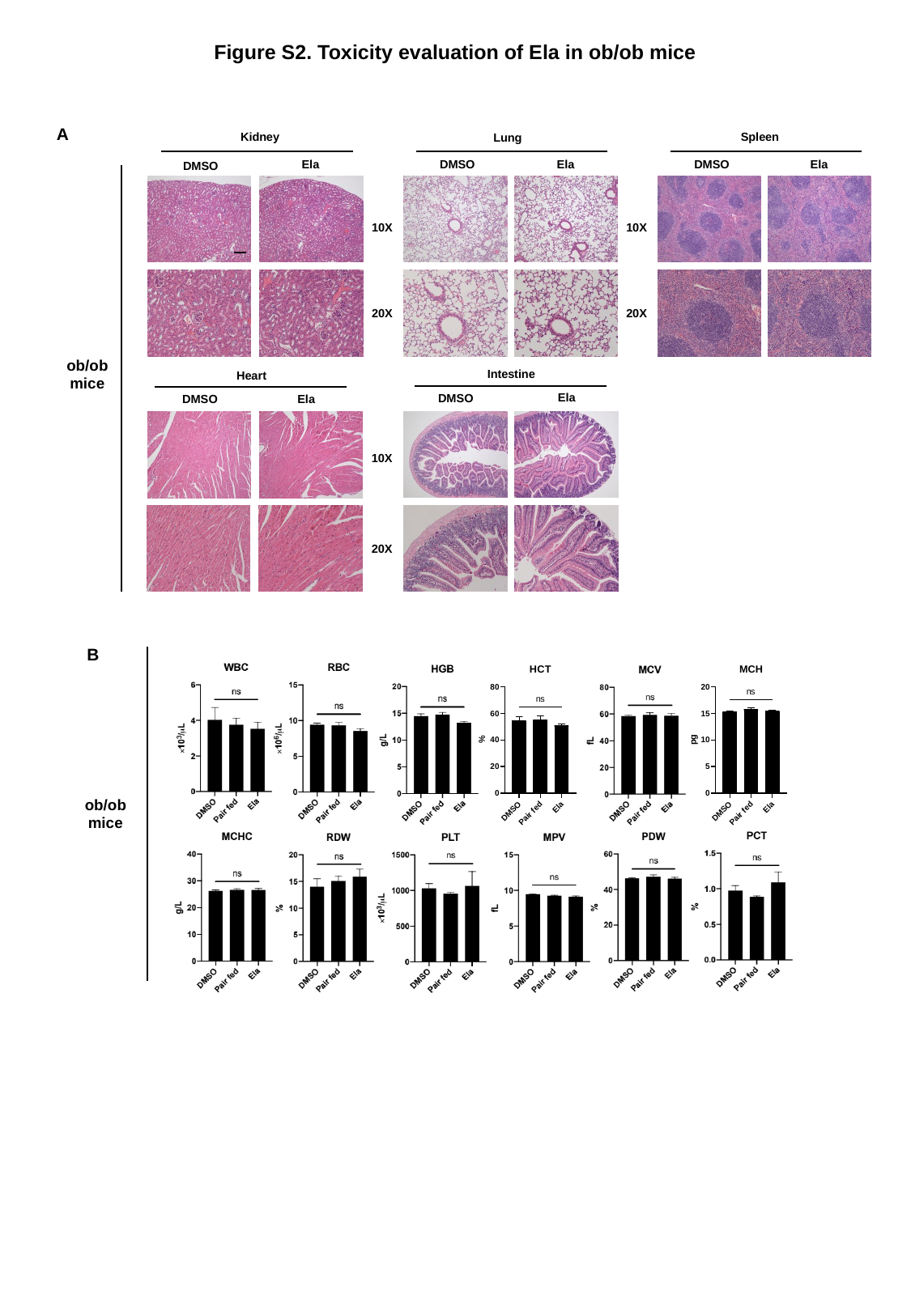

Figure S2. Toxicity evaluation of Ela in ob/ob mice
A
Spleen
Kidney
Lung
Ela
Ela
Ela
DMSO
DMSO
DMSO
10X
10X
20X
20X
ob/ob
mice
Intestine
Heart
Ela
DMSO
Ela
DMSO
10X
20X
B
ob/ob
mice

## Slide 5
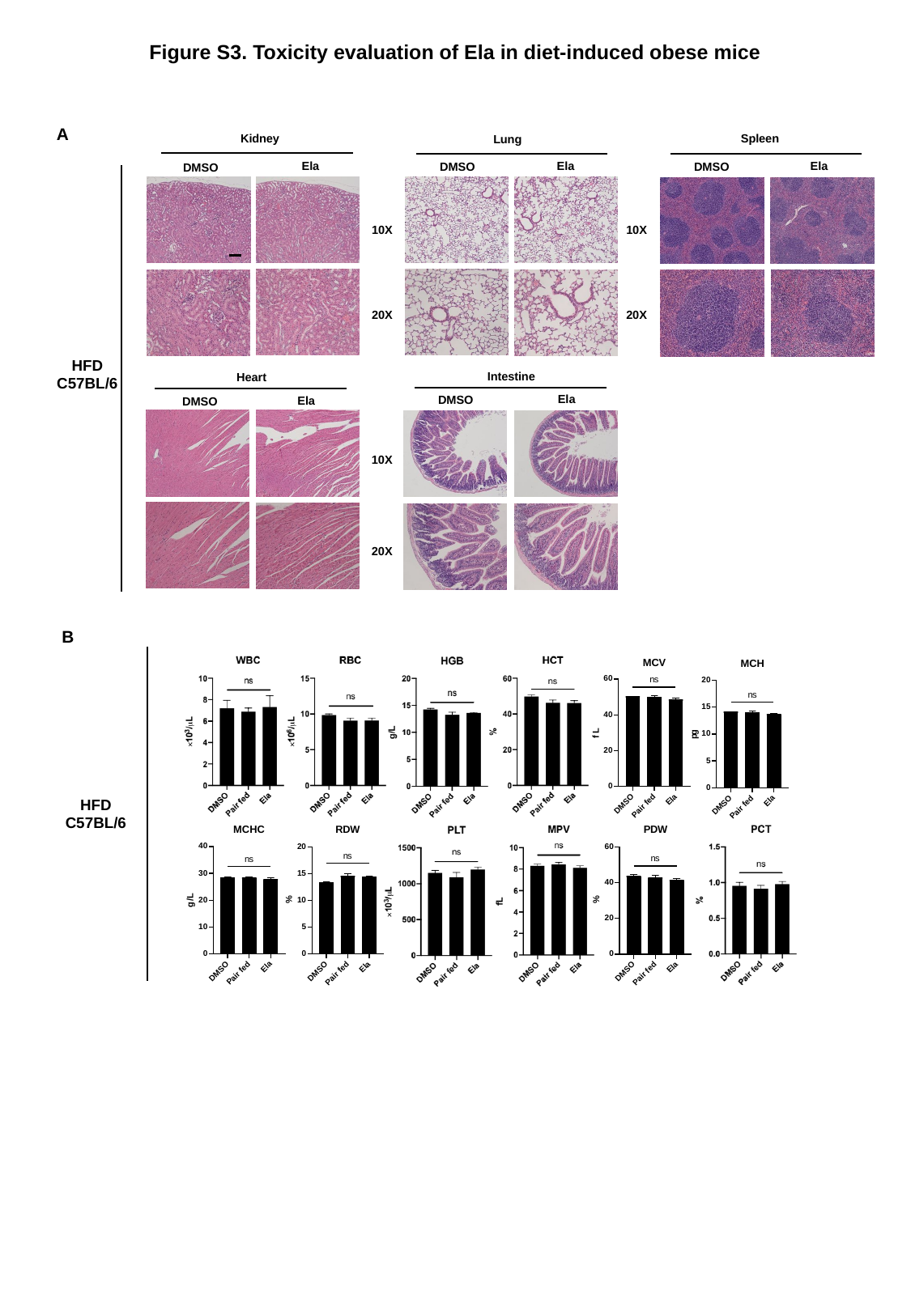

Figure S3. Toxicity evaluation of Ela in diet-induced obese mice
A
Spleen
Kidney
Lung
Ela
Ela
Ela
DMSO
DMSO
DMSO
10X
10X
20X
20X
HE staining
Kidney, lung, heart, spleen, intestine
HFD
C57BL/6
Intestine
Heart
Ela
DMSO
Ela
DMSO
10X
20X
B
HFD
C57BL/6

## Slide 6
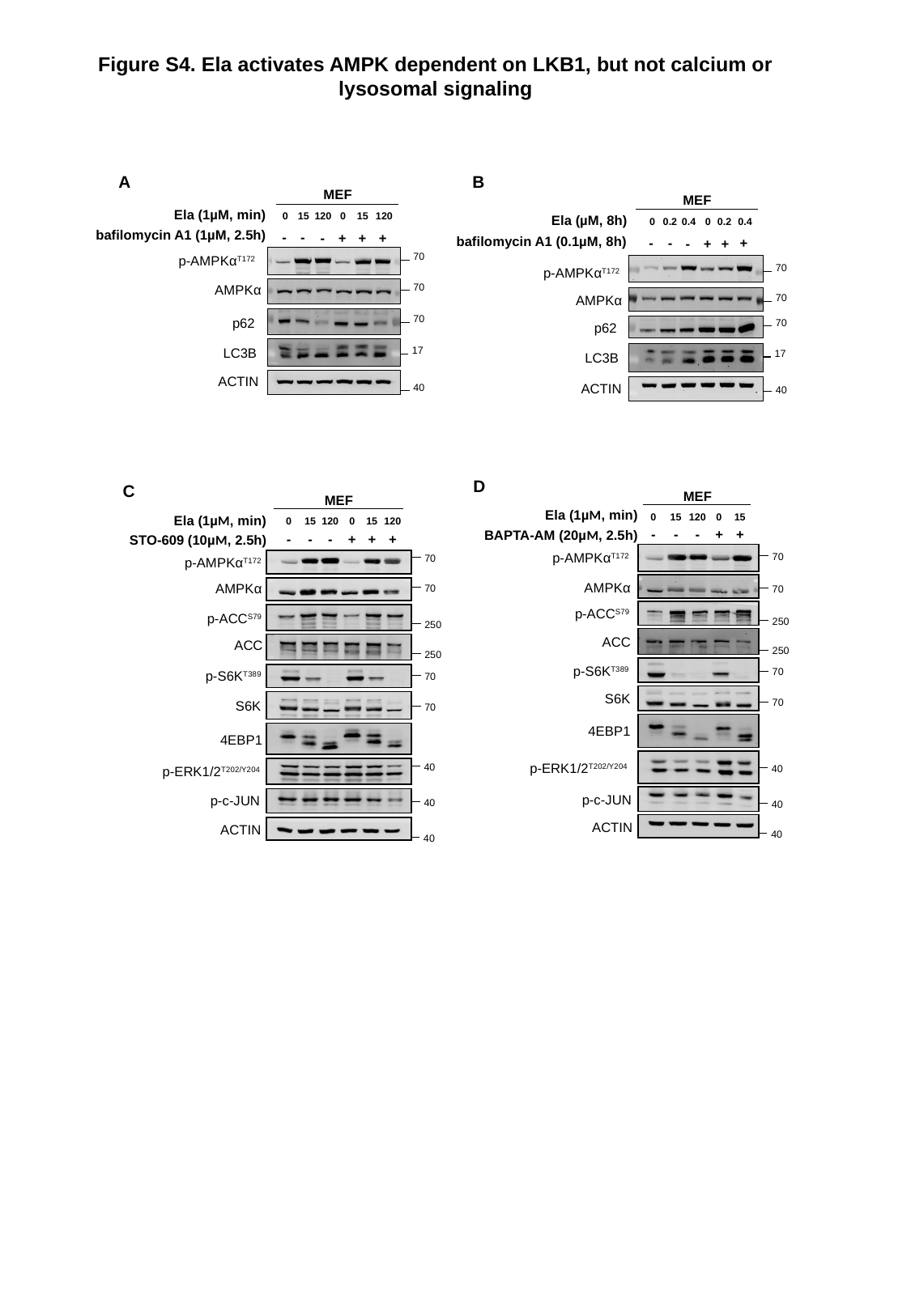

Figure S4. Ela activates AMPK dependent on LKB1, but not calcium or lysosomal signaling
A
MEF
Ela (1µM, min)
15
15
0
120
0
120
bafilomycin A1 (1µM, 2.5h)
-
-
-
+
+
+
70
p-AMPKαT172
70
AMPKα
70
p62
17
LC3B
ACTIN
40
B
MEF
Ela (µM, 8h)
0.2
0.2
0
0.4
0
0.4
bafilomycin A1 (0.1µM, 8h)
-
-
+
-
+
+
70
p-AMPKαT172
70
AMPKα
70
p62
17
LC3B
ACTIN
40
D
MEF
Ela (1µM, min)
0
15
120
0
15
-
-
-
+
+
BAPTA-AM (20µM, 2.5h)
p-AMPKαT172
70
AMPKα
70
p-ACCS79
250
ACC
250
p-S6KT389
70
S6K
70
4EBP1
p-ERK1/2T202/Y204
40
p-c-JUN
40
ACTIN
40
C
MEF
Ela (1µM, min)
0
15
120
0
15
120
-
-
-
+
+
+
STO-609 (10µM, 2.5h)
70
p-AMPKαT172
AMPKα
70
p-ACCS79
250
ACC
250
p-S6KT389
70
S6K
70
4EBP1
40
p-ERK1/2T202/Y204
p-c-JUN
40
ACTIN
40

## Slide 7
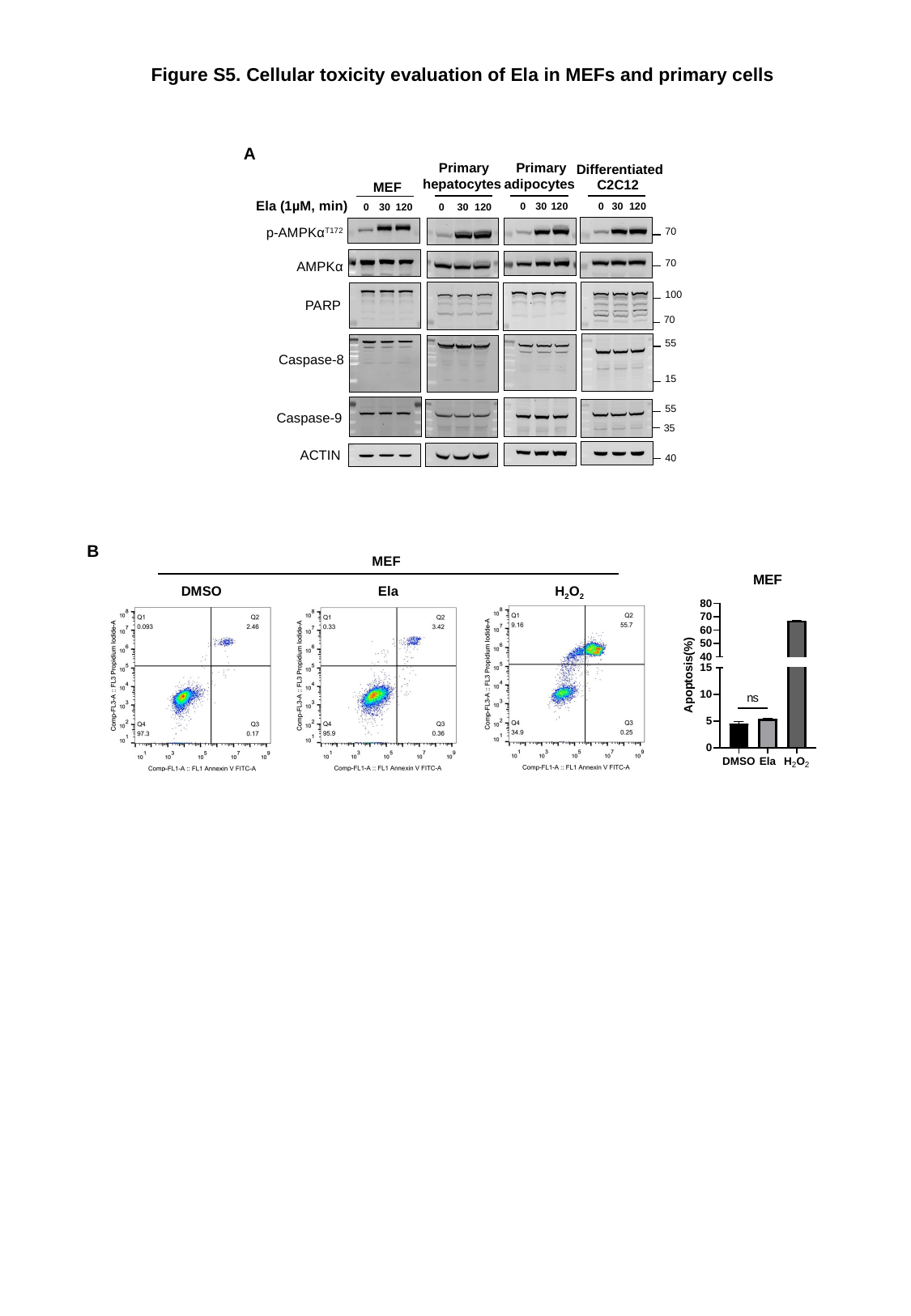

Figure S5. Cellular toxicity evaluation of Ela in MEFs and primary cells
A
Primary
hepatocytes
Primary
adipocytes
Differentiated
C2C12
MEF
Ela (1µM, min)
0
30
120
0
30
120
0
30
120
0
30
120
p-AMPKαT172
70
70
AMPKα
100
PARP
70
55
Caspase-8
15
55
Caspase-9
35
ACTIN
40
B
MEF
H2O2
Ela
DMSO

## Slide 8
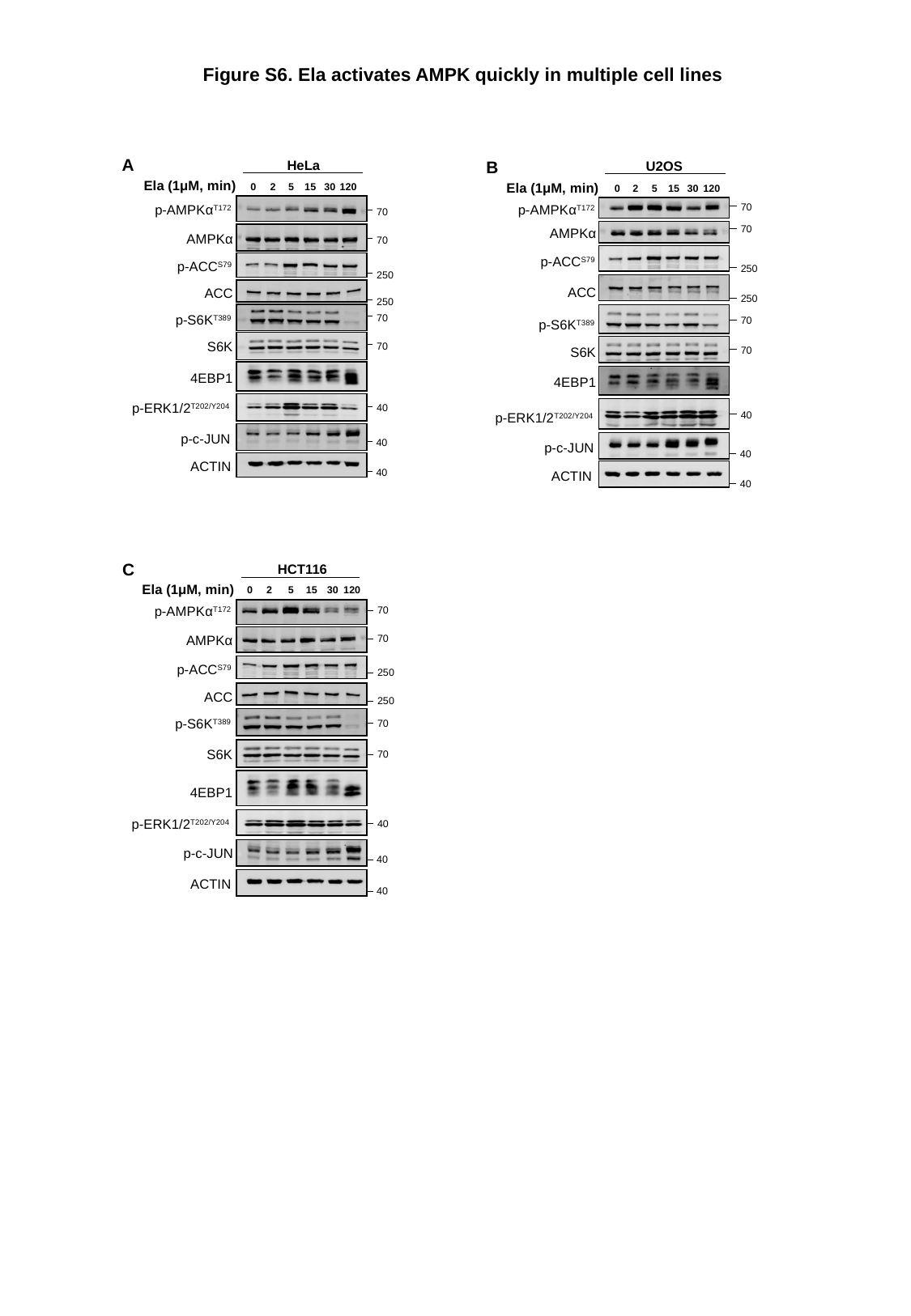

Figure S6. Ela activates AMPK quickly in multiple cell lines
A
HeLa
Ela (1μM, min)
0
2
5
15
30
120
p-AMPKαT172
70
AMPKα
70
p-ACCS79
250
ACC
250
p-S6KT389
70
S6K
70
4EBP1
p-ERK1/2T202/Y204
40
p-c-JUN
40
ACTIN
40
B
U2OS
Ela (1μM, min)
0
2
5
15
30
120
70
p-AMPKαT172
70
AMPKα
p-ACCS79
250
ACC
250
70
p-S6KT389
70
S6K
4EBP1
40
p-ERK1/2T202/Y204
p-c-JUN
40
ACTIN
40
C
HCT116
Ela (1μM, min)
0
2
5
15
30
120
p-AMPKαT172
70
70
AMPKα
p-ACCS79
250
ACC
250
p-S6KT389
70
S6K
70
4EBP1
p-ERK1/2T202/Y204
40
p-c-JUN
40
ACTIN
40
